# Berberine Inhibits Abdominal Aortic Aneurysm Formation and Vascular Smooth Muscle Cell Phenotypic Switching by Regulating the Nrf2 Pathway

**DOI:** 10.1111/jcmm.70509

**Published:** 2025-04-07

**Authors:** Sanjun Li, Xiaoyong Xiao, Yuechen Chang, Ziyao Xu, Xianping Zheng, Haiwen Zhou, Haiqiang Ding, Weiling Lu, Tian Li, Yu Tao

**Affiliations:** ^1^ Department of Cardiology Jiangxi Provincial People's Hospital, the First Affiliated Hospital of Nanchang Medical College Nanchang China; ^2^ Department of Emergency Medicine The First Affiliated Hospital of Shenzhen University, Health Science Center, Shenzhen Second People's Hospital Shenzhen Guangdong China; ^3^ Experimental Center of Medical School of Shihezi University Shihezi China; ^4^ Senior Department of General Surgery The First Medical Center of Chinese PLA General Hospital Beijing China; ^5^ Intensive Care Unit Zibo Central Hospital Zibo China; ^6^ Department of Cardiology Guangdong Cardiovascular Institute, Guangdong Provincial Key Laboratory of Prevention and Treatment of Coronary Heart Disease，Guangdong Provincial People's Hospital (Guangdong Academy of Medical Sciences), Southern Medical University Guangzhou China; ^7^ Tianjin Key Laboratory of Acute Abdomen Disease‐Associated Organ Injury and ITCWM Repair Institute of Integrative Medicine of Acute Abdominal Diseases, Tianjin Nankai Hospital, Tianjin Medical University Tianjin China

**Keywords:** aortic aneurysm, berberine, Keap1, Nrf2, vascular smooth muscle cell

## Abstract

Abdominal aortic aneurysm (AAA) is a life‐threatening disease featuring extensive membrane destruction in the vascular wall, which is closely associated with the phenotypic switching of vascular smooth muscle cells (VSMC). A thorough understanding of the changes in regulatory factors during the pathogenesis of VSMC phenotypic switching is essential for medical treatments in AAA. NRF2 was deemed to hold a pivotal position in developing AAA, especially as it can regulate VSMC phenotypic switching. In this study, we found that berberine prevents the formation of AAA by regulating the phenotypic switching of VSMC, which was well validated in both in vitro and in vivo functional experiments. Mechanically, we found that berberine regulates VSMC phenotypic switching by promoting the expression of downstream VSMC contraction genes through the deubiquitination of Keap1, in which the deubiquitinating enzyme USP15 plays an important mediating role in this process.

## 1 Introduction

The most dangerous type of vascular disease is called an abdominal aortic aneurysm (AAA), defined as the balloon‐like dilatation of blood vessels in the abdominal aorta. To meet the criteria, the diameter of the expanded aorta expands to more than 1.5 times its normal diameter [1]. The fatal characteristics of AAA are hidden onset and sudden rupture, which lead to sudden death [2]. At present, surgical repair is the main treatment method for abdominal aortic aneurysms, and there is no obvious drug that can effectively control the growth rate of AAA or reduce the risk of aortic aneurysm rupture [3]. One important component of the aortic mediators, contractile vascular smooth muscle cell (VSMC), are responsible for maintaining vascular tone [4]. Dysfunction of VSMCs can give rise to various vascular pathologies, such as atherosclerosis [5], hypertension [6], intimal hyperplasia [7], and pulmonary hypertension [8]. Under the action of injury, vascular inflammation and oxidative stress, VSMCs will dedifferentiate, proliferate, migrate, and weaken their contractility, forming a synthetic phenotype, which is the fundamental cause of the injury of the aorta wall and one of the main causes of the occurrence of AAA [9]. Therefore, studying the mechanism of phenotypic transformation of VSMCs and effective intervention measures has become the focus of AAA research.

The cytoplasm contains the master cellular sensor for oxidative stress, Nrf2, which binds to Keap1 [10]. When the cell is exposed to external stimuli, the active site of Keap1 undergoes oxidation, which prevents Keap1 from interacting with Nrf2. As a result, Nrf2 can translocate into the nucleus [11]. Nrf2 moves to the nucleus and attaches itself to antioxidant response elements (AREs), which activate downstream genes, including HO‐1 and NQO1. This induces oxidative stress, leads to calcification, and transforms the phenotype of VSMC [12]. Studies have reported that NRF2 can protect the progression of AAA by influencing the phenotypic switching of VSMC [13], so drugs developed for NRF2 have significant clinical value for the treatment of AAA [14, 15].

**FIGURE 1:**
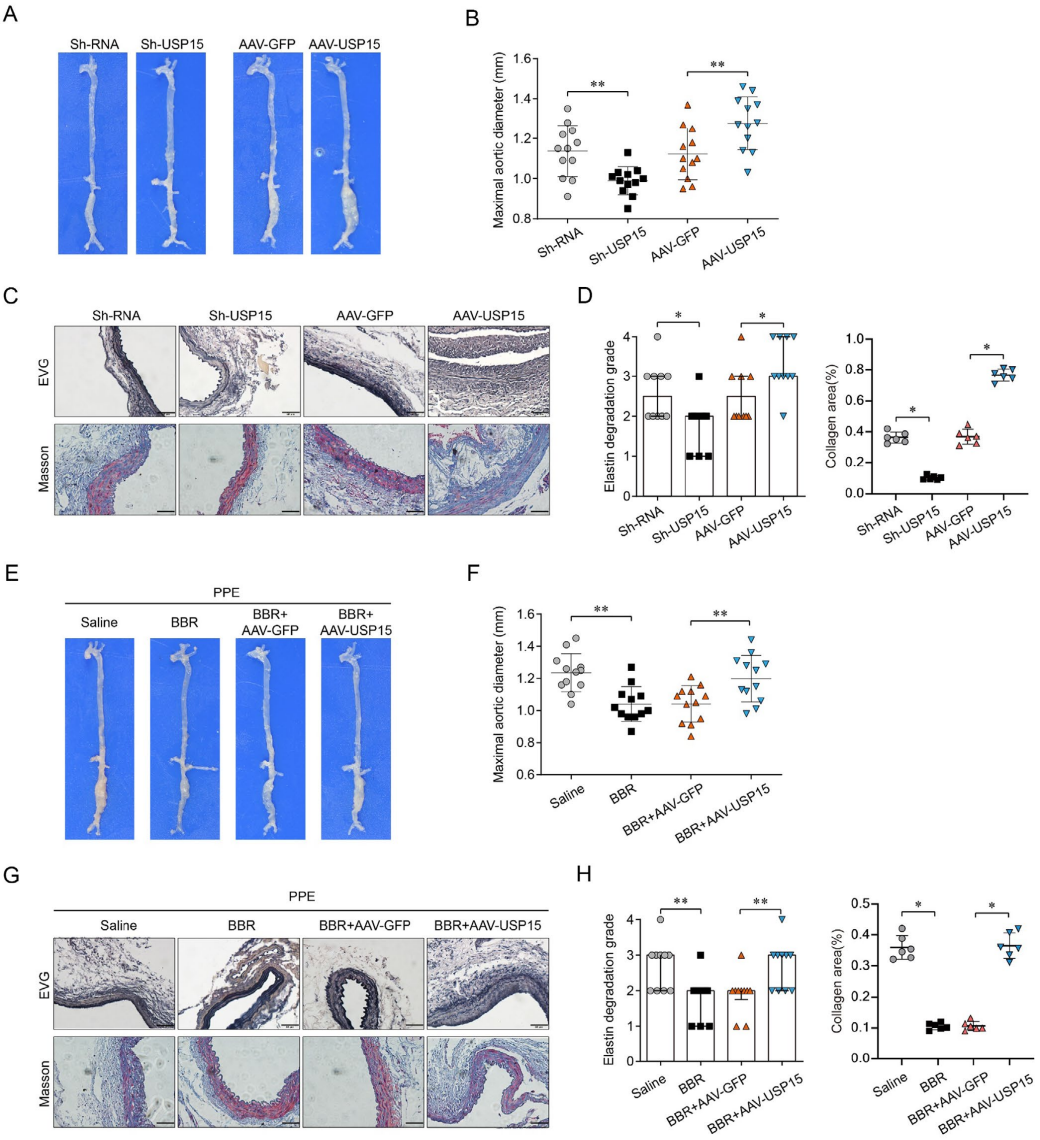
Graphical signal. Exogenous addition of berberine combines with USP15. The reduced level of USP15 leads to the degradation of KEPA1, which in turn leads to a decrease in the degradation of NRF2. NRF2 binds to the promoters of VSMC contraction marker genes and promotes their expression, ultimately inhibiting the formation of AAA.

An isoquinoline alkaloid known as berberine (Brb) is widely found in several Chinese medicinal plants, including barberry, Chinese Coptis, and the roots, rhizomes, and bark of Canadian hydrated plants [16]. Studies have shown that both humans and animals have low bioavailability of brb. Despite low bioavailability, Brb has shown great effectiveness in treating many diseases. Brb has various valuable biological and pharmacological effects, such as antioxidant [17], anti‐inflammatory [18], antidiabetic [19], antitumor [20], antimicrobial [21], hepatoprotective [22], and anti‐dyslipidemia [23]. In recent years, Brb has been found to exhibit biological activities in cardiovascular disorders, including hypertension and ischemic heart disease [24, 25, 26]. Previous studies also demonstrated clear linkages between Brb and NRF2 pathways, and that the Nrf2 signalling pathway specifically mediates the therapeutic and biological activities of Brb [27, 28, 29]. Brb can play a series of effects, such as being anti‐inflammatory and antioxidant, to treat cardiovascular diseases. However, the role of Brb in AAA is unclear, so we investigated whether berberine might influence the development of AAA through NRF2‐mediated regulation of VSMC phenotypic switching (Figure 1).

## 2 Materials and Methods

### 2.1 Animals

We acquired male C57BL/6J mice, aged 10 to 12 weeks, from Southern Medical University's Laboratory Animal Center. The mice were maintained in a pathogen‐free condition with a consistent temperature (22°C), humidity (60%–65%), and a 12‐h light/dark cycle. They were provided with water and the normal chow rodent meal. The Institutional Animal Care and Use Committee protocols of Southern Medical University were followed when conducting all animal experiments.

### 2.2 PPE‐Induced Murine AAA Model

The PPE‐induced AAA model was implemented as described in the previous study [30]. 10–12 weeks old mice were anaesthetised by inhaling 1.5%–2.0% isoflurane. The mice were then randomly assigned to distinct groups. After the disappearance of the pedal withdrawal reflex, the infrarenal abdominal aorta was exposed through a midline incision of the lower abdomen and incubated with a sterile gauze soaked with PPE or inactive PPE (pre‐heated at 100°C for 30 min) for 5 min. Then, the gauze was removed, and the abdominal cavity was triple rinsed with sterile saline before closing the abdominal cavity. Two weeks after PPE exposure, the mice were sacrificed for further experiments.

### 2.3 Berberine Treatment

After the implementation of the AAA model, mice were fed berberine (40 mg/kg/d) daily by gavage for in vivo examinations. The controls were given the same volume of saline. For in vitro experiments, cells were pre‐incubated with 100 μM berberine for 1 h. An equivalent volume of sterile saline was used for the pre‐incubation of the control cells.

### 2.4 Aneurysm Quantification

An overdose of anaesthetics euthanised the mice, and the chest and abdomen cavities were opened to expose the aorta completely. Before isolation from the mice, the aortas were perfused with 4°C phosphate‐buffered saline (PBS; Gibco, CA, USA) at physiological pressure through a left ventricular puncture to remove the blood. After the surrounding tissues were decorticated, the aortas were photographed using an electronic video microscope (DZ‐5980; Nreeohy, Guangdong, China). The infrarenal abdominal aorta was regarded as the part between the right renal artery and the bifurcation of the bilateral common iliac arteries. The maximal outer diameter of the infrarenal aorta was measured at the most prominent position of dilation. The dilation in the outer width of the infrarenal aorta by 50% or greater compared with that in mice treated with inactive PPE was defined as aneurysm formation [31].

### 2.5 Mouse Primary VSMC Isolation and Culture

According to the prior studies, male C57BL/6J mice aged 10–12 weeks were used to isolate mouse primary aorta VSMCs [32]. In brief, aortas were harvested from mice after the surrounding adipose tissues were removed. Then, in Hank's balanced salt solution (HBSS; Gibco, CA, USA) at 37°C for 10 min, the aortas were digested in 1 mg/mL type II collagenase, 0.744 U/mL elastase, and 1 mg/mL trypsin inhibitor. After full removal of the adventitial layer, the aortas were cut longitudinally, and the intimal layer was softly scraped off. Then, the aortas were cut into 1 × 1 mm pieces and further digested in the digestive fluid at 37°C for 2 h. Dulbecco's Modified Eagle Medium/Nutrient Mixture F‐12 (DMEM/F‐12; Gibco, CA, USA) was added at an equivalent volume, which contained 20% fetal bovine serum (FBS; Gibco, CA, USA), in order to terminate the digestive reaction. After centrifuging at 300×*g* for 5 min, the cells were collected and resuspended in DMEM/F‐12 supplemented with 10% FBS. After that, the cells were cultivated for adhesion for 48 h. VSMCs' passages 4–7 were employed for further examinations.

### 2.6 VSMC Transfection

Mouse VSMCs were incubated for 6 h with 50 nM siRNAs or the control group using Lipofectamine RNAiMAX Reagent (Invitrogen, CA, USA) in accordance with the manufacturer's guidelines to facilitate the transfection of small interfering RNAs (siRNAs). The sequences of the siRNAs against USP7, USP15, USP16, USP25, BAP1, and OTUD1, and the corresponding controls are provided in Table S1. For overexpression of USP15, mouse VSMCs were incubated with 4 μg pcDNA3.1‐USP15 (oe‐USP15) or the control plasmids (oe‐NC) for 24 h.

### 2.7 Mouse Adeno‐Associated Virus Transfection

Mice were injected with 2 × 10^11^ genomic copies of adeno‐associated viruses (AAVs) through tail veins to knock down or overexpress USP15. The transfection was performed 2 weeks before the AAA model establishment.

### 2.8 RNA Extraction and Quantitative Real‐Time Polymerase Chain Reaction

In adherence to the manufacturer's guidelines, total RNAs were isolated from tissues or cells utilising the TRIzol reagent (Invitrogen, CA, USA). After determining concentrations, the extracted RNAs were reverse transcribed into cDNAs using the PrimeScript RT reagent Kit manufactured by TaKaRa in Shiga, Japan. Using the LightCycler 480 II equipment from Roche in Basel, Switzerland, a quantitative real‐time polymerase chain reaction (qPCR) was carried out using the SYBR Green RT‐PCR Kit from Yeasen Biotechnology in Shanghai, China. The primer sequences utilised in our analysis can be found in Table S2. The mRNA levels were normalised using the housekeeping gene glyceraldehyde‐3‐phosphate dehydrogenase (GAPDH) and then measured using the 2−ΔΔCT^−△△Ct^ method.

### 2.9 Immunohistochemistry

The aortas were isolated and immersed in a 4% paraformaldehyde solution for 24 h. Afterward, they were embedded in paraffin. The specimens were sliced into serial sections with a thickness of 5 μm and a spacing of 500 μm. The paraffin was then removed, and the sections were rehydrated by treating them with xylene, 100% ethanol, 90% ethanol, 70% ethanol, and distilled water. The sections were subjected to antigen retrieval for 15 min by boiling them in citrate buffer (pH 6.0), following which the endogenous peroxidase activity was quenched by subjecting them to another treatment with 3% hydrogen peroxide. The nonspecific binding sites were blocked for an hour at room temperature using 2% bovine serum albumin (BSA; Beyotime, Shanghai, China). Subsequently, the sections were incubated overnight at 4°C with the primary antibodies in 1% BSA, followed by 30 min at room temperature with horseradish peroxidase—labelled streptavidin (Beyotime, Shanghai, China). Haematoxylin was used to counterstain the sections, which were stained with diaminobenzidine. The primary antibodies used are shown in Table S3. Immunohistochemical results were quantified by counting the integration optical density (IOD) values of the positive staining area with Image‐Pro Plus software 6.0 (Media Cybernetics, Md., USA). At least five sections were measured to determine the average value of each mouse.

### 2.10 Elastin Van Gieson Staining

Paraffin sections were stained according to the manufacturer's instructions using the Collagen Fibre and Elastic Fibre Staining Kit (Solarbio, Beijing, China). As previously described, a standard for semi‐quantification of elastin degradation was introduced: score one, no degradation; score two, slight elastin degradation; score three, severe elastin degradation; score four, complete fracture of elastic fibres [33].

### 2.11 Western Blot

Protease inhibitor cocktail and phosphatase inhibitor (Thermo Fisher Scientific, MA, USA) were added to RIPA buffer (Thermo Fisher Scientific, MA, USA) to facilitate the extraction of proteins from tissues or cells. The proteins were then electrophoresed on 10% SDS‐PAGE running gels and transferred onto polyvinylidene fluoride (PVDF) membranes. The membranes were blocked for 2 h at 37°C with 5% BSA in Tris‐Buffered Saline Solution with Tween‐20 (TBST). The samples were incubated with primary antibodies for an additional hour at 4°C, followed by secondary antibodies conjugated with horse peroxidase for 1 h at room temperature (Beyotime, Shanghai, China). The proteins were identified utilising enhanced chemiluminescence (ECL; Beyotime, Shanghai, China), and the results were quantified using ImageJ software (National Institutes of Health, Bethesda, MD, USA). Each experiment was done at least three times.

### 2.12 Molecular Dynamics Simulation

AMBER16 software [34] was used to conduct molecular simulation of USP15 and Berberine, and the best conformation obtained by docking Autodock‐vina was used as the initial conformation of the molecular dynamics simulation. Before the simulation began, AMBER16 was used to optimise the small molecule structure, and Antechamber was used to calculate the ligand RESP9 charge. The complex structure of the ligand and receptor protein was placed in A regular octahedral cube box with a thickness of 12 Å in the solvent layer. The TIP3P water model [35] was filled with FF14SB [36] force field for the protein receptor and gaff2 force field for the ligand. Na^+^ ion is added as a counterbalancing ion to make the system reach a neutral state. The energy of the system is minimised by using the maximum reduction algorithm, and the chemical bonds connected with hydrogen atoms are rigorously constrained by the SHAKE method during the 100 PMD simulation, keeping the bond length and bond Angle unchanged. Set the simulation time step to 2 fs. After the initial minimisation, the entire system was heated from 10 to 300 K at a constant volume during the simulation, and 100 ns unconstrained molecular dynamics simulations were performed under the canonical ensemble (NVT) and isothermal isobaric ensemble (NPT) and repeated three times. In this study, periodic boundary conditions (PBC) were used to eliminate the edge effect of the solvent box. In addition, the Robo‐Mesh Ewald (PME) algorithm is used to calculate the long‐term electrostatic interaction with a truncation value of 1.2 nm. The CPPTRAJ module in AMBER16 is used for simulation trajectory analysis. The entire simulation process supports CUDA8.0 and GPU‐accelerated work.

### 2.13 Statistical Analysis

A p‐value of < 0.05 was deemed statistically significant for all of the study's statistical tests. The Shapiro Wilk test was first performed for continuous variables to confirm whether the variables correspond to normal distribution. Analyses of normally distributed data were conducted using the unpaired Student's *t*‐test for two independent groups, and one‐way ANOVA with a post‐Bonferroni's multiple comparisons test was utilised for multiple groups. The Mann–Whitney *U* test, a nonparametric method for independent groups, was used to analyse non‐normal data. We used Fisher's exact test to compare AAA incidences, and for survival analyses between groups, we used the log‐rank (Mantel‐Cox) test. In our study, values are presented as the mean ± SD. The medians and quartiles of the elastin degradation scores are presented. GraphPad Prism 6.01 (GraphPad Software, CA, USA) and SPSS software 20.0 (IBM Corporation, NY, USA) were utilised for statistical analyses.

## 3 Results

### 3.1 Brb Inhibits the Formation of Abdominal Aortic Aneurysms

**FIGURE 2:**
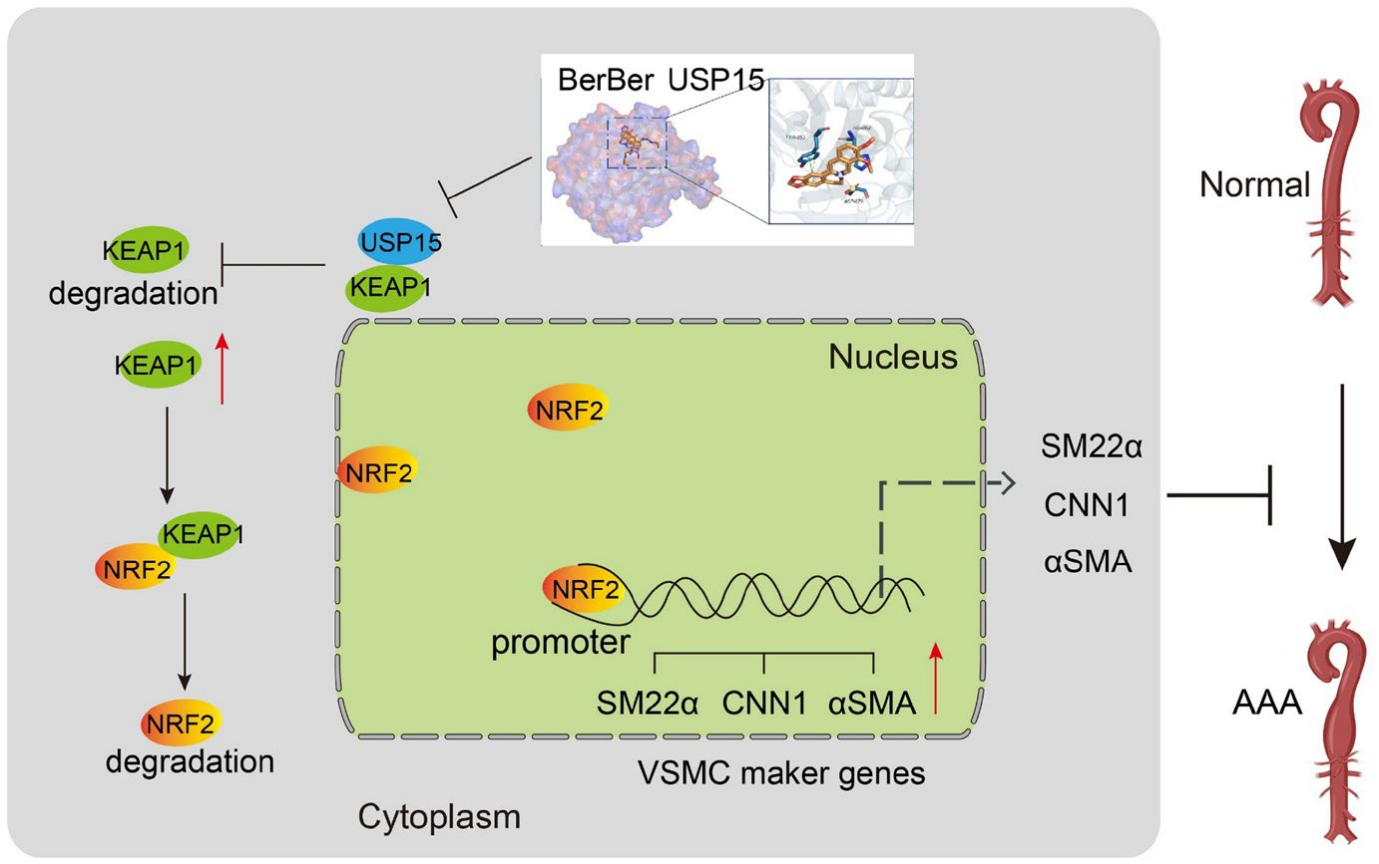
Effect of Brb on AAA formation and VSMC phenotypic switching in mice. (A, B). The AAA was established in PPE‐induced mouse AAA mode, and the maximum abdominal aortic diameter of AAA mice was significantly reduced in the Brb treatment group (*p* < 0.05). (C, D). Elastic fibre and collagen destruction in the blood vessels was improved in PPE‐induced mouse AAA mode，which can be reversed by Brb treatment (*p* < 0.05). (E). WB protein expression analysis revealed that the PPE model group's VSMC tissues had significantly higher levels of classical contraction factors (CNN1, SM22, α‐SMA) than the PPE‐inactivated group's (*p* < 0.05). (F). WB protein expression analysis showed that the Brb group could inhibit the expression levels of CNN1, SM22 and α‐SMA in the PPE model in human primary VSMC (*p* < 0.05).

A PPE‐induced mouse AAA model was developed in order to verify the effect of Brb on AAA formation. The maximal abdominal aortic ratio in the PPE mouse model group was significantly higher than in the control group, but there was a significant decrease in the maximal abdominal aortic ratio by injecting mice with Brb intravenously (Figure 2A,B). Identical collagen degradation and elastic fibre destruction, which quantification by elastin degradation grade in the PPE mouse model group was notably aggravated compared to the control group, but there was a notable decrease in collagen degradation and elastic fibre destruction by injecting mice with Brb intravenously (Figure 2C,D).

### 3.2 Brb Inhibits the Phenotypic Switching of VSMC

Recently, increasing evidence has shown that VSMC phenotypic switching played an important role in the development of AAA and that the switching from contraction phenotype to synthetic phenotype was an early event of AAA. It has been reported that Brb regulates the phenotypic switching of VSMC; therefore, we hypothesised that Brb influences the progression of AAA through this mechanism. The WB protein expression analysis revealed that the PPE model group's VSMC tissues had considerably lower levels of classical contraction factors (CNN1, SM22 and α‐SMA) than the PPE‐inactivated group. The Brb group could significantly improve the PPE model's CNN1, SM22, and α‐SMA expression levels. This result was confirmed in mouse models and human primary VSMC (Figure 2E,F). This analysis showed that Brb plays an important regulatory role in the phenotypic switching of VSMC in AAA.

### 3.3 Brb Inhibits AAA by Mediated Expression of Keap1/NRF2

**FIGURE 3:**
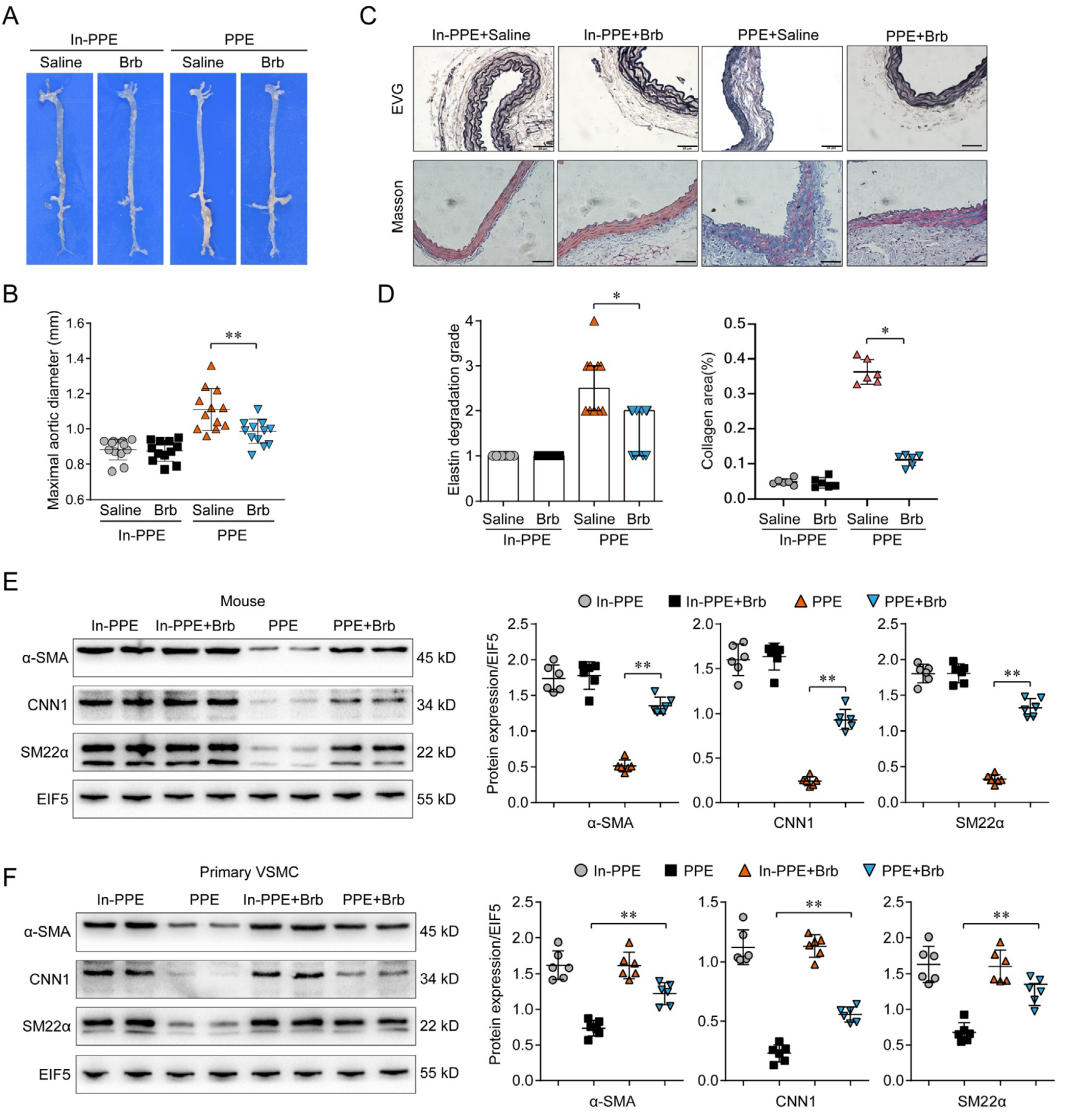
The effects of Brb and USP15 on the expression level of NRF2. (A) WB protein expression analysis showed that NRF2 expression in primary VSMCs was very low under normal conditions but significantly increased after PPE stimulation, while NRF2 was significantly increased in the Brb treatment group, Keap1 levels were lower in the PPE group and even lower after Brb treatment (*p* < 0.05). (B) WB protein expression analysis showed that the longer the PPE was added, the lower the NRF2 expression; the changing trend remained the same even after Brb treatment, and the trend of Keap1 expression remained consistent, as described above (*p* < 0.05). (C) PCR expression analysis showed that USP7, USP15, and USP25 were significantly increased in AAA, with USP15 having the highest expression (*p* < 0.05). (D) WB protein expression analysis showed that interference with USP15 led to a further decline in Keap1 expression and an increase in NRF2 expression after PPE stimulation on the primary VSMC (*p* < 0.05). (E) WB protein expression analysis showed that after PPE stimulation of primary VSMC, even after Brb treatment, overexpression of USP15 still resulted in decreasing NRF2 expression level (*p* < 0.05).

According to previous studies, NRF2 can contribute to the phenotypic switching of VSMC and performs a key protective role in the AAA process. Some studies have also reported that Brb can regulate the phenotypic switching of VSMC through NRF2. In order to clarify the specific role, we verified in vitro experiments, which showed that NRF2 expression in VSMC was very low under normal conditions but significantly increased after PPE stimulation, while NRF2 was significantly increased in the Brb treatment group. Keap1 levels were lower in the PPE group and even lower after Brb treatment, suggesting that Brb can promote NRF2 expression (Figure 3A). However, further experiments found that the longer the PPE was added, the lower the NRF2 expression; the changing trend remained the same even after Brb treatment, and the trend of Keap1 expression remained consistent as described above (Figure 3B). Previous studies have shown that under normal physiological conditions, Keap1 can promote the degradation of NRF2 protein and maintain the expression of NRF2 protein at a low level, but under pathological stimulation, Keap1 expression decreases and NRF2 expression increases. Our study found that Brb can promote the degradation of Keap1, thereby inhibiting the degradation of NRF2 protein, suggesting that Brb can stabilise the NRF2 protein level by promoting the degradation of Keap1. The above results suggested that Brb may inhibit AAA formation by mediating the expression of NRF2.

### 3.4 USP15 Mediates the VSMC Phenotypic Switching by Keap1/NRF2

Previous literature reported that several ubiquitination‐related genes could regulate the degradation of Keap1, including USP7, USP15, USP16, USP25, BAP1, and OTUD1, among which the expressions of USP7, USP15, and USP25 were significantly different in AAA and significantly up‐regulated in AAA. We detected the expression of ubiquitination genes RNA in AAA arterial tissue and showed that USP7, USP15, and USP25 were significantly increased in AAA, with USP15 having the highest expression (Figure 3C). However, the specific roles and mechanisms of USP7, USP15, and USP25 in regulating AAA remain unclear, especially whether they participate in Brb regulation of VSMC phenotypic switching. As mentioned above, we found that after PPE stimulation of VSMC, the expression of Keap1 decreased, and NRF2 increased compared with the non‐intervention group. It was detected that interference with USP15 led to a further decline in Keap1 expression and an increase in NRF2 expression after PPE stimulation on the primary VSMC, while the trend of other ubiquitination genes was less pronounced (Figure 3D). Further experiments showed that after PPE stimulation of primary VSMC, even after Brb treatment, overexpression of USP15 still resulted in decreasing NRF2 expression level(Figure 3E). Therefore, USP15 is likely a key ubiquitination gene in Brb‐mediated NRF2 expression.

### 3.5 The Molecular Dynamics Simulation Between USP15 and Brb

**FIGURE 4:**
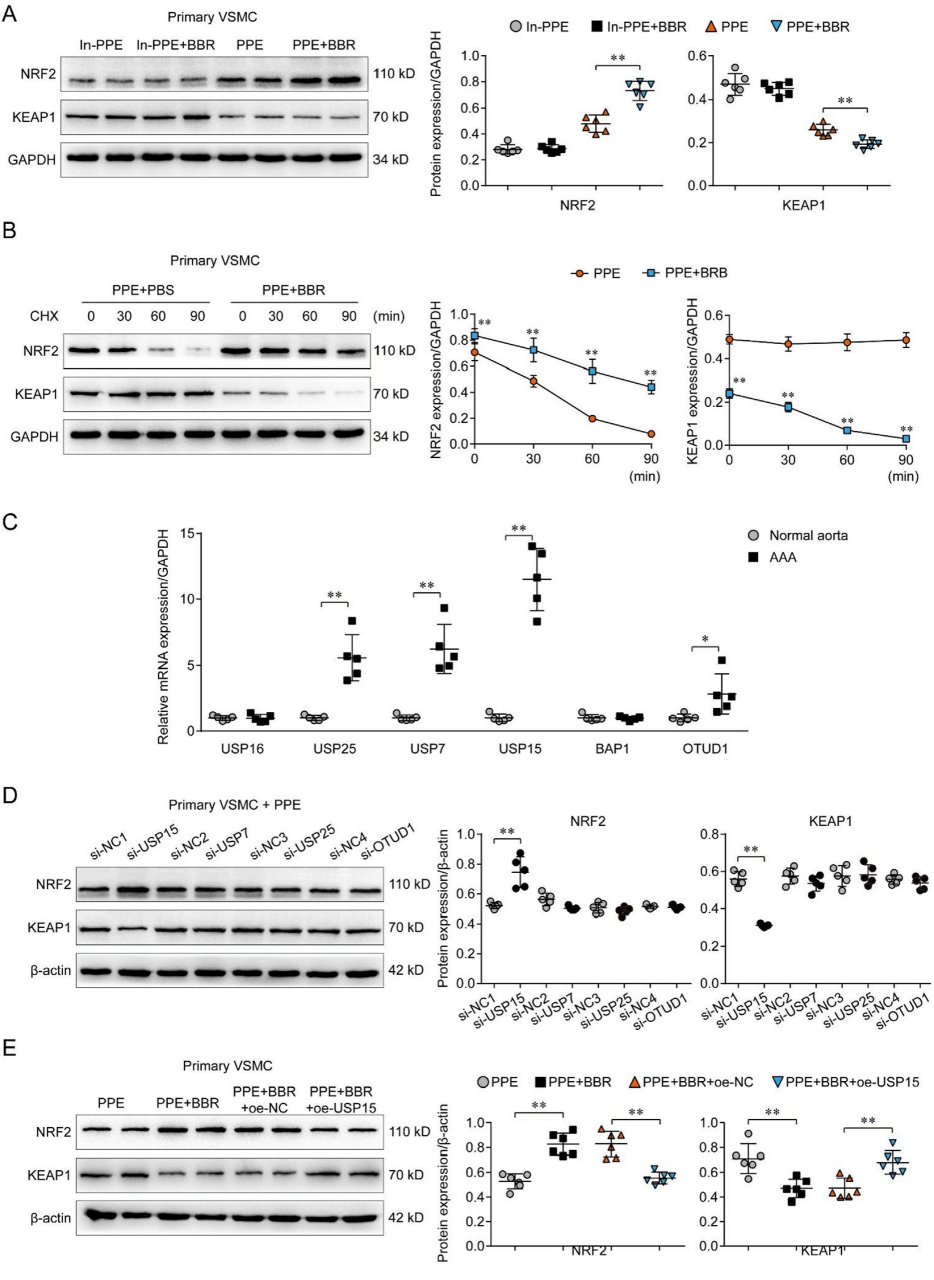
100 ns molecular dynamics simulation diagram of Brb and USP15. (A) USP15‐Berberine complex structure diagram. (B) RSMD between berberine and USP15 protein. (C) RSMF between berberine and USP15 proteins. (D) SASA between berberine and USP15 proteins. (E) The convolution between berberine and USP15 proteins.

In order to determine the degree of correlation between USP15 and berberine, we next conducted the molecular dynamics simulation analysis. Root mean square deviation (RMSD) is a good measure of the conformational stability of proteins and ligands, and also a measure of the degree of deviation of the atomic position from the starting position. The smaller the deviation, the better the conformational stability. We analysed the change of RMSD value of the USP15 –rberine complex (Figure 4A). As shown in Figure 4B, RMSD of the USP15—rberine fluctuates in the early stage and becomes stable after 30 ns. Compared with RMSD of the empty protein, the fluctuation of berberine after binding with usp15 is smaller, indicating that small molecules can bind usp15 stably. The RMSF curve shows the fluctuation of amino acid residues in a protein. It can be seen from Figure 4C that residues near the binding site after small molecules bind to usp15 exhibit lower RMSF values. In combination with the molecular simulation of RMSD and RMSF, we believe that the binding effect of USP15 and Berberine is good, so we have carried out in‐depth analysis of the USP15—rberine complex with multiple indexes. Solvent accessible surface area (SASA) is a measure of the surface area of a protein. We determined the SASA (Figure 4D) between USP15 and Berberine. The results showed that the decrease of SASA after the binding of small molecules to usp15 indicated that the protein and small molecules were tightly bound. The radius of rotation curve (Rog) indicates the density of the overall structure of the protein. It can be seen from Figure 4E that the ROG value of the protein decreases after the combination of small molecules, indicating that the structure of the protein becomes more compact and stable.

### 3.6 The Effects of USP15 and Brb on the Expression Level of VSMC Contraction Marker Genes

**FIGURE 5:**
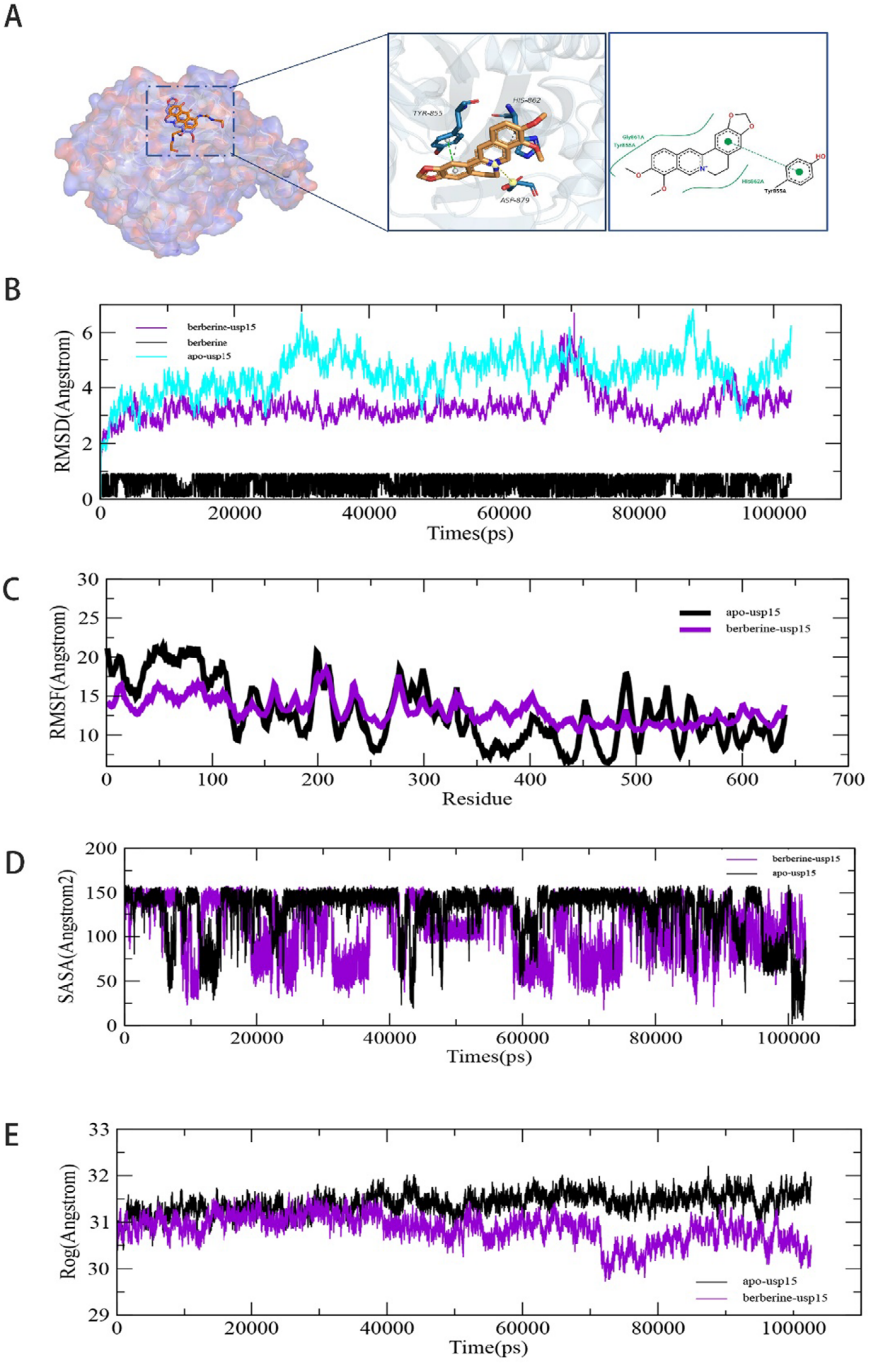
The effects of USP15 and Brb on the expression level of VSMC contraction marker genes. (A, B). WB protein expression analysis showed that interference with USP15 could lead to decreased expression of PPE‐induced contraction marker genes in VSMC, while the result was the opposite after overexpression of USP15 (*p* < 0.05). (C). Further rescue experiments showed that after USP15 overexpression, the promoting effect of Brb on VSMC contraction marker genes was weakened (*p* < 0.05).

To determine whether USP15 is involved in Brb regulation of VSMC phenotypic switching, we performed Brb remediation of USP15 in the PPE mouse model. First, western blot results showed that the expression of contractile factors was significantly increased after USP15 knockdown by VSMC in the control group. PPE‐induced decreases in VSMC contraction factors expression were offset after knocking down USP15 in VSMC (Figure 5A). Thereafter, the expression of contractile factors did not show a significant change after USP15 overexpression in the control group, but PPE‐induced decreases in VSMC contraction factors expression were further decreased after USP15 overexpression in VSMC (Figure 5B).

Further rescue experiments showed that after USP15 overexpression, the promoting effect of Brb on VSMC contraction marker genes was weakened (Figure 5C). These results suggest that USP15 is an important intermediate link in the regulation of VSMC phenotypic switching by Brb.

### 3.7 Brb Inhibits the Formation of AAA by Mediated USP15

**FIGURE 6:**
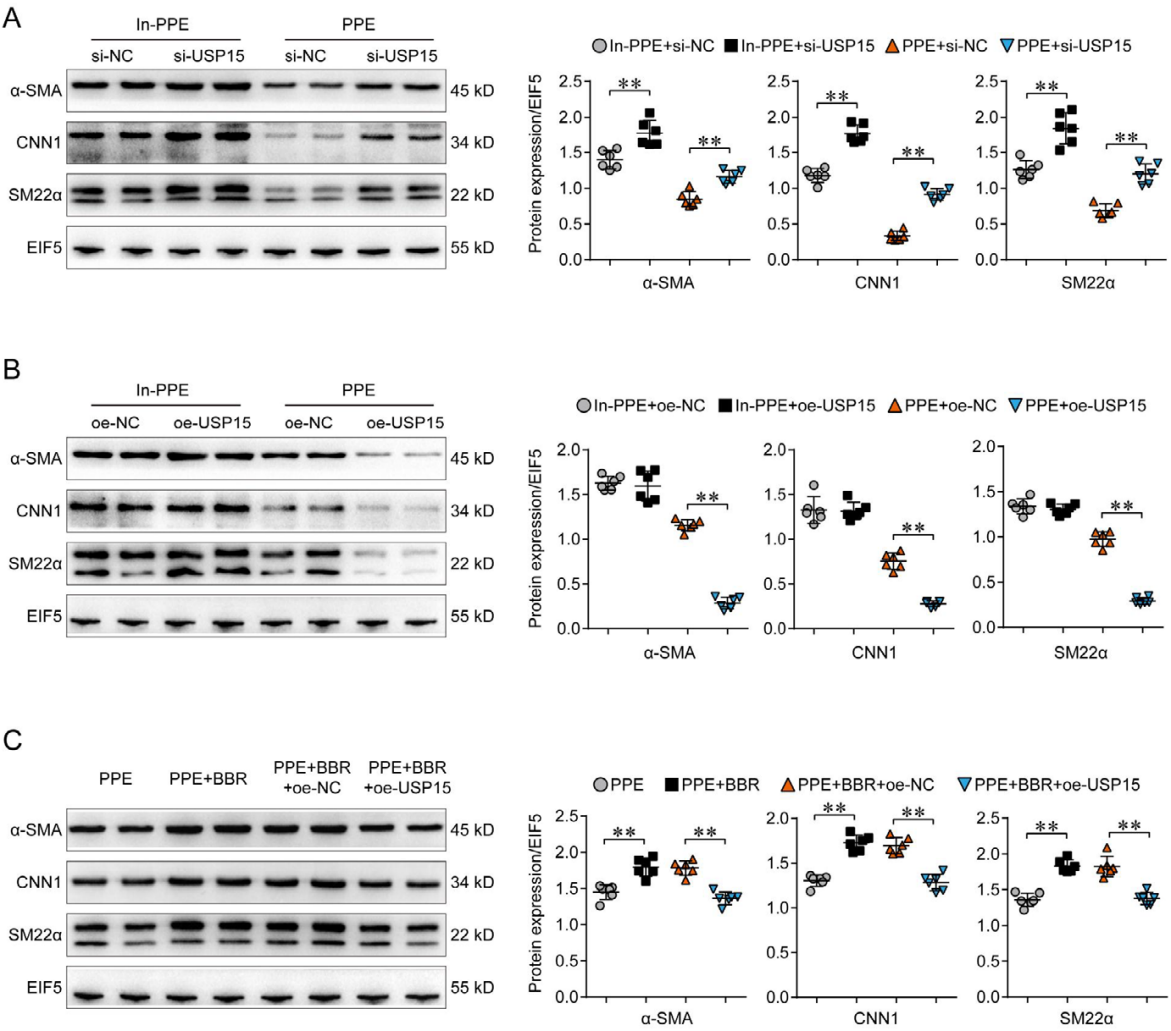
Effect of USP15 on AAA formation in mice. (A–D). Interference with USP15 significantly decreased the maximum abdominal aortic diameter of AAA mice, and the degradation of collagen and the destruction of elastic fibres in the blood vessels were improved, while the results of overexpression of USP15 were opposite (p < 0.05, scale bars = 50 μm in C). (E, F). Vivo rescue experiments showed that the maximum abdominal aortic diameter of AAA mice was significantly decreased after treatment with Brb alone in the PPE‐induced mouse AAA model. However, after overexpression of USP15 on this basis, the maximum abdominal aortic diameter was not significantly improved (*p* < 0.05). (G, H). The change trend of degradation of collagen and the destruction of elastic fibres in the blood vessels were same as maximum abdominal aortic diameter above (*p* < 0.05).

In order to verify the effect of USP15 on AAA, the PPE‐induced mouse AAA model was first constructed, and the effect of interference and overexpression of USP15 on the formation of AAA in mice was observed, and the maximum abdominal aortic diameter and vascular remodelling of mice were detected. The results showed that interference with USP15 significantly decreased the maximum abdominal aortic diameter of AAA mice, and the degradation of collagen and the destruction of elastic fibres in the blood vessels were improved, while the results of the overexpression of USP15 were opposite (Figure 6A–D). These results indicate that USP15 expression in mice can significantly accelerate the occurrence and development of AAA and can enhance the inflammatory response of vascular tissue.

Further, vivo rescue experiments showed that the maximum abdominal aortic diameter of AAA mice was significantly decreased after treatment with Brb alone in the PPE‐induced mouse AAA model. However, on this basis of USP15 overexpression, the maximum abdominal aortic diameter was not significantly improved (Figure 6E,F). The change trend of degradation of collagen and the destruction of elastic fibres in the blood vessels was the same as the maximum abdominal aortic diameter above (Figure 6G,H). Therefore, we can assume that Brb mediates the occurrence and development of AAA through USP15, and the important mechanism is NrF2‐mediated phenotypic switching.

## 4 Discussion

### 4.1 Main Interpretation

Cardiovascular diseases remain the main killer worldwide [37, 38, 39, 40, 41, 42]. Among these, we found that berberine prevents the formation of AAA by regulating the phenotypic switching of VSMC. Mechanically, we found that berberine regulates VSMC phenotypic switching by promoting the expression of downstream VSMC contraction genes and also inhibiting the expression of inflammatory factors through the Nrf2 pathway, in which the deubiquitinating enzyme USP15 plays an important mediating role in this process. Our findings suggest that berberine may be a safe and inexpensive treatment for AAA.

An essential cellular mechanism for responding to oxidative stress‐induced damage is the KEAP1‐NRF2‐ARE (Kelch‐like ECH‐associated protein, Nuclear factor erythroid 2‐related factor 2, Antioxidant response element) signalling pathway [12]. NRF2 can regulate a series of expressions of antioxidant genes through its binding to the promoter containing the antioxidant reaction element ARE. Over the past few decades, a deeper understanding of NRF2 has been gained, and it has been found to play an important role in many fields, such as cancer, neurodegenerative diseases, and metabolic diseases [43, 44, 45]. In recent years, studies have suggested that Nrf2 has an obvious protective function in the cardiovascular system [46]. Studies have also shown that Nrf2 can maintain cardiovascular health by regulating the function of vascular smooth muscle cells, anti‐inflammation, anti‐aging, and phenotypic switching [47, 48]. NRF2 is expressed at a low level under normal physiological conditions, but it is significantly increased under stress, and the ubiquitination mechanism is an important factor leading to this phenomenon. Keap1, a substrate adaptor of Cullin3 (Cul3)‐dependent E3 ubiquitin ligase complex, facilitates the ubiquitination of NRF2. Keap1‐CUL3‐E3 is a functional E3 ubiquitin ligase complex that can be formed with Cul3 and Rbx1 [49]. Keap1 comprises three functional domains: the Kelch or DGR domain, the IVR domain, and the BTB domain. Keap1 dimerization requires the BTB domain because it binds Cul3. The ETGE and DLG motifs mentioned above are crucial for preserving the interaction between Nrf2 and Keap1 and can be interacted with by the Kelch/DGR domain. Connecting the BTB and Kelch/DGR domains, the IVR also comprises cysteine residues that control the activity of Keap1. Keap1‐Cul3‐E3 ubiquitin ligase promotes the ubiquitination of multiple lysine residues in the Neh2 domain, found at the N terminal of Nrf2 (between the DLG and ETGE motifs), under typical physiological circumstances. The ubiquitinated targets are subsequently transported to the 26S proteasome for degradation. Previous research has shown that NRF2, which is significantly overexpressed in AAA, may prevent the disease from progressing by regulating the phenotypic switching of VSMCs [13]. Brb is the main active ingredient in Coptis, which has a long history of pharmacological effects in China and plays an important role in hypoglycaemia, hypolipidaemia, anti‐tumour, and anti‐cardiovascular diseases. This is the first study to show that Brb can stabilise NRF2 levels via USP15 expression, thereby regulating the expression of the VSMC contraction gene under stress and facilitating NRF2 entry into the nucleus, thereby preventing the development and occurrence of AAA.

Based on previous studies, the efficacy of Brb in prevention and treatment is determined mainly by its influence on the Nrf2 signalling pathway. Studies on the process of Brb antioxidation have revealed that inactivation of the NRF2 signalling route can reduce the protective effect of Brb, suggesting that the Nrf2 signalling pathway is essential to this process. Brb's ability to function as an antioxidant primarily comes from how it affects the Nrf2 signalling pathway, strengthening the body's defences against oxidative damage and antioxidants. In addition, studies on other diseases (diabetes, ischemic heart disease) have found that Brb can reduce the production of ROS and mitochondrial damage by stimulating the Nrf2 signalling pathway to achieve the therapeutic effect of the above diseases while silencing the expression of NRF2 counteracts the therapeutic effect of Brb on diseases. Consequently, the NRF2 signalling pathway serves as a crucial mediator that mediates the role of Brb. However, the specific mechanism of how Brb affects NRF2 in exerting its role in this pathway is unclear. Given the previously reported important role of the ubiquitin‐proteasome pathway in regulating stress response by strictly regulating NRF2, we hypothesised that Brb may regulate NRF2 through deubiquitinating enzymes to protect AAA progression. In this study, it was found that Brb could inhibit the expression of the deubiquitinating gene USP15, promote NRF2 expression, and thus drive the expression of VSMC contraction genes and inhibit the expression of inflammatory factors. The ubiquitin‐proteasome system is a multi‐step process involving many different proteins. The proteasome recognises and localises these ubiquitin‐labelled proteins (peptides), which subsequently undergo degradation. Cells can specifically and accurately degrade excess proteins through various programmed intensive reaction processes. As one of the main regulatory proteins of the ubiquitin‐proteasome system, USP family proteins have been previously reported to be involved in regulating several biological pattern changes in cells. This is highly compatible with the occurrence and progression of various diseases. In addition, studies have shown that the USP family proteins are key regulatory molecules in the ubiquitin‐proteasome system, which can be important in the onset and progression of cardiovascular diseases (such as ischemic cardiomyopathy and aortic aneurysm/dissection). A member of the USP family, USP15 is extensively expressed in various tissues and controls various cellular functions. Studies have shown that USP15 can selectively damage leukaemia progenitor cells by participating in homeostasis REDOX reaction while preserving normal haematopoietic function, thus playing a role in the treatment of AML, and this process is closely related to the inhibition of intracellular KEAP1 protein and NRF2. However, there have been few reports on the role of USP15 in regulating cardiovascular diseases, and the upstream and downstream mechanisms of USP15 and NRF2 regulation remain unclear. Initially, we discovered that USP15 regulates the progression of AAA via the NRF2 signalling pathway and that the upstream target drug Brb may have therapeutic potential for AAA via the USP15/NRF2 signalling axis.

### 4.2 Limitations

There are still some limitations in this study. First, the molecular biological mechanism of how berberine regulates USP15 is still unclear and needs to be further verified by subsequent experiments. Second, animal experiments in vivo did not confirm the role of USP15 in regulating Nrf2 by Brb in abdominal aortic aneurysm. Third, Brb is limited to the basic experimental level, and its role in treating AAA in clinical patients can be explored in the future.

### 4.3 Conclusion

In conclusion, this study found that berberine may play a role in treating AAA through the NRF2 signalling pathway, which has not been reported in previous studies. Moreover, our study has important clinical significance and scientific value in the search for drugs to treat AAA.

## Author Contributions

**Sanjun Li:** writing – original draft (equal). **Xiaoyong Xiao:** validation (equal). **Yuechen Chang:** data curation (equal), writing – review and editing (equal). **Ziyao Xu:** data curation (equal), writing – review and editing (equal). **Xianping Zheng:** data curation (equal), writing – review and editing (equal). **Haiwen Zhou:** visualization (equal). **Haiqiang Ding:** visualization (equal). **Weiling Lu:** methodology (equal). **Tian Li:** writing – review and editing (equal). **Yu Tao:** writing – review and editing (equal).

## Consent

All authors have agreed to publish this manuscript.

## Conflicts of Interest

The authors affirm that they do not possess any conflicting interests.

## Supporting information


Table S1.

## Data Availability

Data are available at https://www.jianguoyun.com/p/DR1vuxIQuaiFChiAvdYFIAA.
