# Supplementary material for: Berberine Inhibits Abdominal Aortic Aneurysm Formation and Vascular Smooth Muscle Cell Phenotypic Switching by Regulating the Nrf2 Pathway
Source: J Cell Mol Med. 2025 Apr 7;29(7):e70509. doi: 10.1111/jcmm.70509 (PMC11974455; doi:10.1111/jcmm.70509)
Supplement: Supplementary file 1 — Table S1. [file JCMM-29-e70509-s001.docx]

**Supplemental Table 1. siRNA sequences (from 5’ to 3’)**

| si-USP16 | GGAGUGGAAUAUCUGCCAATT |
| --- | --- |
| si-USP25 | GCCUCCAUCAAAUGCUCAATT |
| si-USP7 | GGCCUGCAAUGUUAGAUAATT |
| si-USP15 | GGCCUGCAAUGUUAGAUAATT |
| si-BAP1 | GGAAGAUUUCGGUGUCAAATT |
| si-OTUD1 | CCACUUCAGCCCACUCAUUTT |

**Supplemental Table 2. Primer sequences for quantitative real-time PCR (from 5’ to 3’).**

| GAPDH | Forward | TGTCCGTCGTGGATCTGAC |
| --- | --- | --- |
|  | Reverse | CCTGCTTCACCACCTTCTTG |
| USP16 | Forward | GTTCAAGACGGCAGTGTA |
|  | Reverse | TTCGCATAGGCAGTGTAAT |
| USP25 | Forward | CAGCAGCAAGATGTGAGT |
|  | Reverse | TGAGTTATCGGAGTGTAAGG |
| USP7 | Forward | ACTGGCTATGTCGGCTTA |
|  | Reverse | GCTTATCGCTGTGCTGTA |
| USP15 | Forward | TGGCAGACAACAAGGTATT |
|  | Reverse | GACAAGGTGGCATTCAATC |
| BAP1 | Forward | TCACCTCTCACATCTCCAA |
|  | Reverse | CTTCCACCACCTCCTTCT |
| OTUD1 | Forward | TACCGATTCCACATCATCC |
|  | Reverse | CAGCATCATAGTGTCCATTG |

**Supplemental Table 3. Antibodies for western blots.**

| Antibody | Vendor or Source | Catalog # | Dilute Proportion |
| --- | --- | --- | --- |
| anti-α-SMA | Abcam | ab32575 | 1/1000 |
| anti-CNN1 | Abcam | ab46794 | 1/5000 |
| anti-SM22α | Abcam | ab14106 | 1/1000 |
| anti-EIF5 | Abcam | ab228874 | 1/1000 |
| anti-β-actin | Abcam | ab5694 | 1/2000 |
| anti-GAPDH | Abcam | ab181602 | 1/10000 |
| anti-NRF2 | Proteintech | 80593-1-RR | 1/1000 |
| anti-KEAP1 | Abcam | ab227828 | /1/2000 |
